# Supplementary material for: DMRforPairs: identifying Differentially Methylated Regions between unique samples using array based methylation profiles
Source: BMC Bioinformatics. 2014 May 15;15:141. doi: 10.1186/1471-2105-15-141 (PMC4046028; doi:10.1186/1471-2105-15-141)
Supplement: Additional file 2 — DMRforPairs output for the comparison of A431-MCF7 and NA17018-NA17105. Please start from the HTML files in each folder. Available via the BMC Bioinformatics website. [file 1471-2105-15-141-S2.zip › 1394847754114233_MOESM2_ESM/A431_MCF7/figures/19312.pdf]

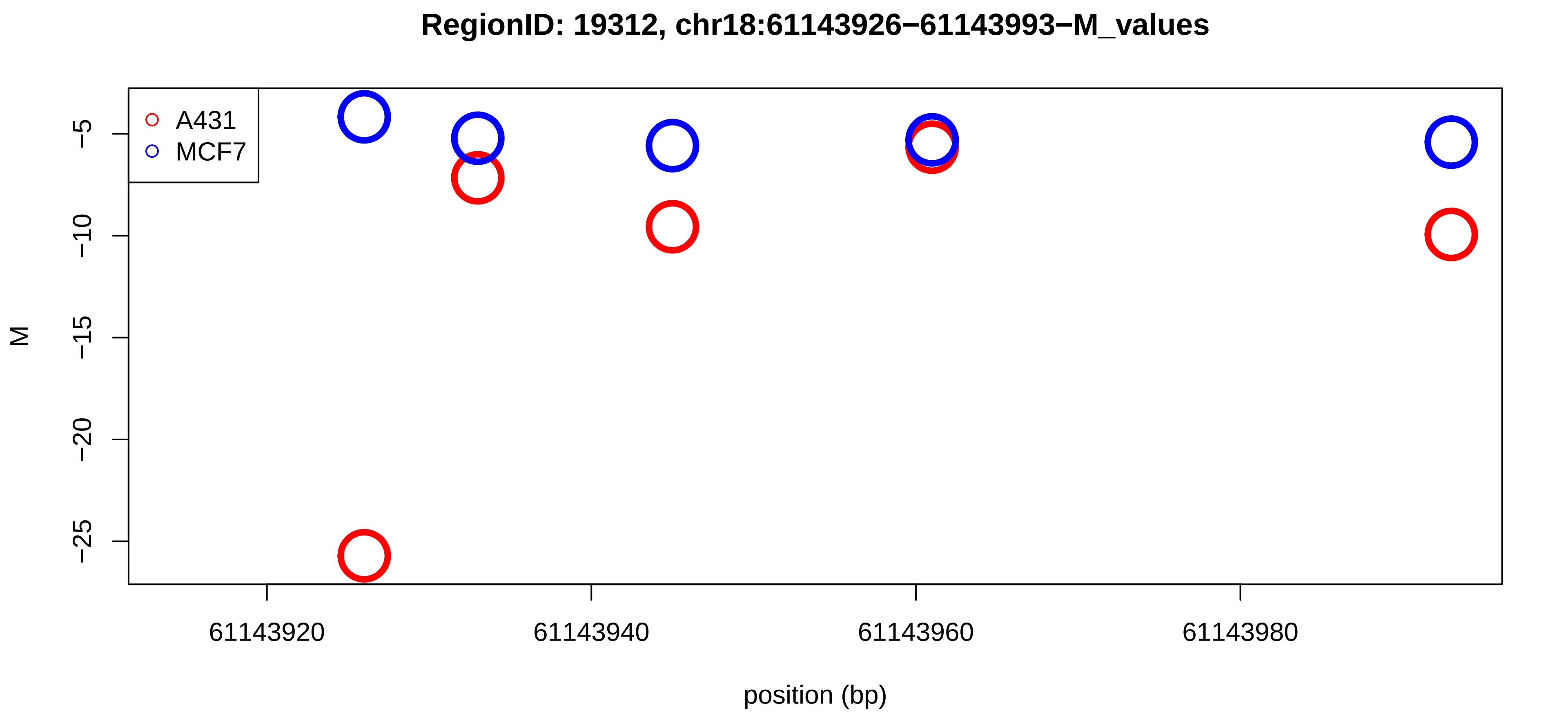
RegionID: 19312, chr18:61143926−61143993−M_values
A431
MCF7
M
−5
−10
−15
−20
−25
61143920
61143940
61143960
61143980
position (bp)

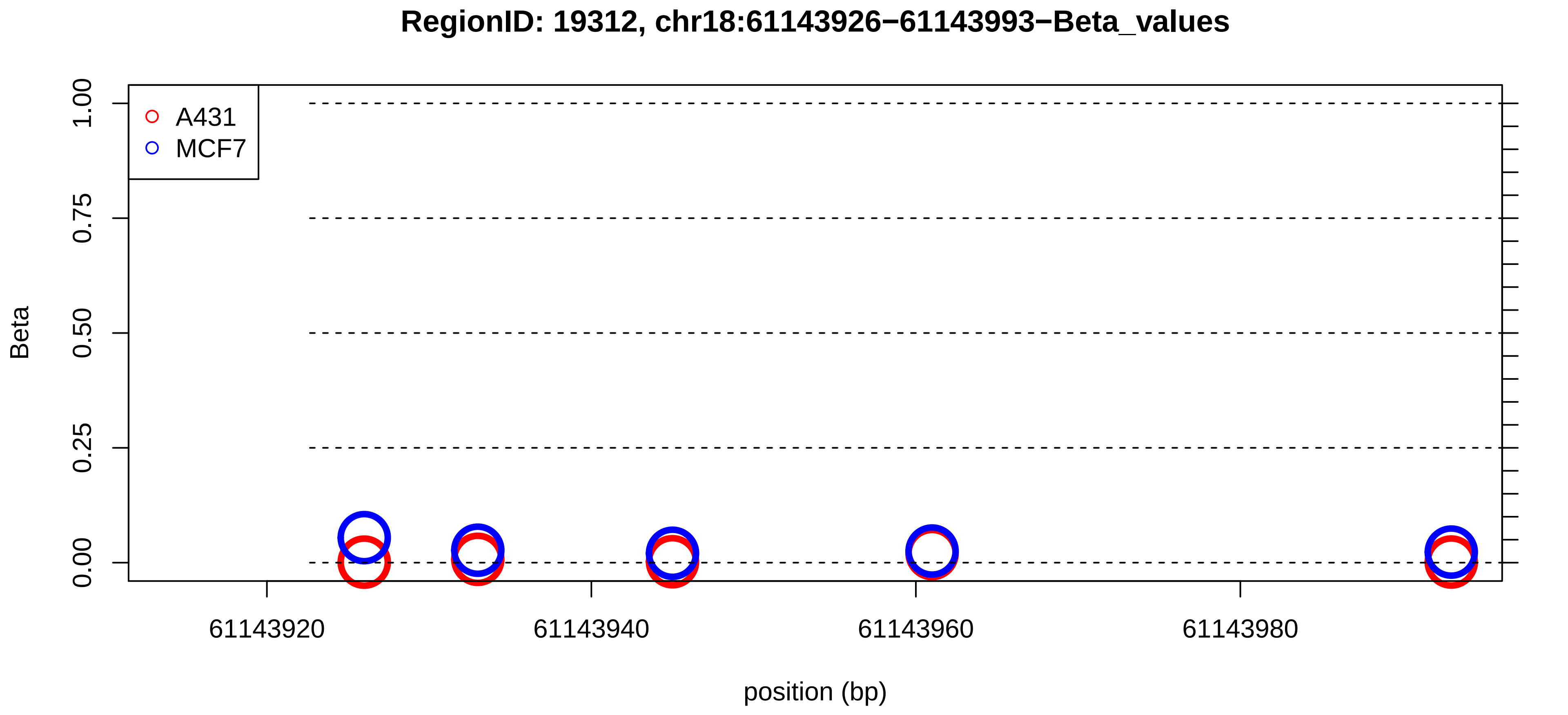
RegionID: 19312, chr18:61143926−61143993−Beta_values
A431
MCF7
Beta
1.00
0.75
0.50
0.25
0.00
61143920
61143940
61143960
61143980
position (bp)

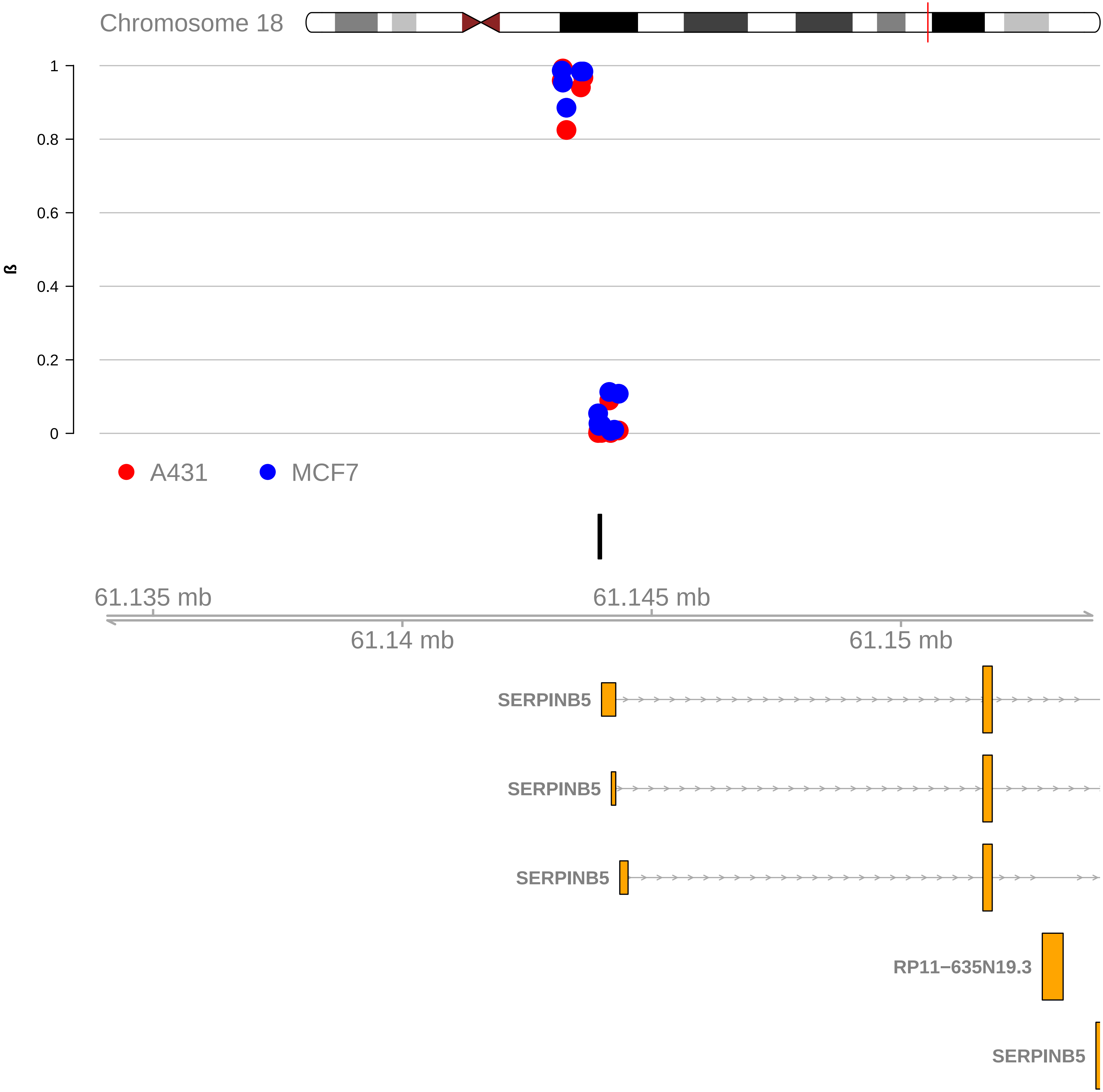
Chromosome 18
ß
1
0.8
0.6
0.4
0.2
0
A431
MCF7
61.135 mb
61.145 mb
61.14 mb
61.15 mb
SERPINB5
SERPINB5
SERPINB5
RP11−635N19.3
SERPINB5
